# Flowering Newsletter 2024

**DOI:** 10.1093/jxb/erae306

**Published:** 2024-08-09

**Authors:** Rainer Melzer

**Affiliations:** School of Biology and Environmental Science and Earth Institute, University College Dublin, Ireland

**Keywords:** Double flowers, FLOWERING LOCUS T, flowering time, fragrance, *Lacandonia*, miR172, *Ranunculales*, rice, rose, *Triticeae*

**Why do we love flowers? Why should everyone love flowers? The answer depends on whom you ask—and when you ask. Ask a freshly married couple on Valentine’s day and it is clear they love flowers for their beauty, their vibrant colours, their captivating scent. Flowers as a symbol of love. Yet, there is a poignant irony in giving cut flowers, which are already destined to die without fulfilling their evolutionary purpose—to set seeds and produce fruits. Which brings us to the second possible answer as to why we love flowers. Ask a botanically inclined divorced couple and it is clear they love flowers for the fruits and their products that bring consolation in difficult times. The barley grains for alcoholic beverages. The cocoa beans for chocolate.**

The articles in this year’s *Flowering Newsletter* broadly fall into those categories: flowers for beauty and flowers for nurture.

We start with Noh *et al.* (2024) who provide a comprehensive review on the fragrance of roses. Over 400 volatile compounds make up the rose fragrance, and the smell of a particular cultivar is determined by the prevalence of one or several of these compounds (Noh *et al.*, 2024). Why do roses smell at all? Probably to attract pollinators, but maybe also to repel herbivores. This proves to be a riddle that awaits resolution: how do you attract some suitors while also deterring those that have damaging effects?

While fragrance is a powerful factor contributing to the beauty of flowers, it is primarily the petals that make them so appealing to humans. More petals are usually equated with more appealing flowers, as is evident by the large number of double flower varieties that exist for many ornamental plants. Some of the first double flower mutants characterized molecularly were the *agamous* (*ag*) mutant in Arabidopsis and the *plena* mutant in *Antirrhinum* (Yanofsky *et al*., 1990; Bradley *et al.*, 1993). Since then, our understanding of gene regulatory networks governing petal numbers has increased considerably and, as outlined by Wang *et al.* (2024) mutations in a number of different pathways can lead to a double flower phenotype. Mutations in the *AG* gene regulatory network may be responsible for the double flower phenotype of many cultivars. It is intriguing, however, that in many cases it is not an *AG* orthologue itself that is mutated. Instead, *APETALA2* (*AP2*) homologues, which constitute *AG* repressors, are expanded in their expression domain. This expansion is caused by *AP2* homologues becoming insensitive to repression by the miRNA *miR172*. Thus, in an interesting twist, the de-repression of a repressor does lead to repression of *AG* expression and an increase in petal numbers (Wang *et al.*, 2024).

The rose fragrance and double flowers are beautiful, but nurture is often provided by the fruits developing from the inconspicuous flowers of cereals: rice and wheat, the staple foods of the world. Zhang *et al*. (2024) explore how understanding the molecular control of inflorescence architecture can help to increase yield in wheat, barley, and related cereals. They identify several genes and gene regulatory circuits that can be used to modify the inflorescence architecture, which in turn may improve grain yield. Intriguingly, *miR172* also features here, as this miRNA and *AP2* homologues are involved in the regulation of inflorescence indeterminacy (Zhang *et al.*, 2024).

The roles of darkness, light, and heat for yield in rice are discussed by Zong *et al*. (2024). The authors illustrate that day length and temperature both affect flowering time in rice and show that temperature and photoperiod are integrated at the molecular level to control flowering time. The review provides specific recommendations for allele combinations promoting or delaying flowering depending on the temperature and day length in particular regions in China (Zong *et al.*, 2024). It will be exciting to watch how this area of research and molecular breeding develops further.

These authors (Zong *et al.*, 2024) also highlight the central importance of *FLOWERING LOCUS T* (*FT*) homologues for flowering time regulation in rice. It is now well established that *FT* encodes florigen, but the quest for florigen lasted decades, and Claire Périlleux recounts the scientific career of Georges Bernier, one of the major figures in flowering time research (Périlleux, 2024). Georges Bernier was the longest serving editor of the *Flowering Newsletter* (from 1989 to 2005) and passed away in 2023. Claire Périlleux paints the picture of a devoted plant scientist and passionate teacher (Périlleux, 2024).

Having talked about beauty and nurture, it appears prudent to remind everyone that some flowers also fall into a third category: intoxicating or even poisonous. This applies particularly to many species of the *Ranunculales* lineage. In the most famous case of tragic love, Romeo ended his life with a poison that may well have been derived from the highly toxic *Ranunculales* monkshood (*Aconitum*). However, as the RanOmics Group outlines, *Ranunculales* have far more to offer than poisonous secondary metabolites (RanOmics Group *et al.*, 2024). The floral and fruit morphology in this group is extraordinarily diverse. Pertinent evolutionary questions such as the evolution of bilateral symmetric flowers, the evolution of nectaries, or the evolution of different fruit types can be addressed using the *Ranunculales* as a model lineage. Ample genomic resources are already available for this lineage, which will pave the way for more exciting discoveries in this important group of flowering plants (RanOmics *et al.*, 2024).

What else did the year provide? As usual, some stunning research on flowers and reproductive development, much of which was covered in the *Journal of Experimental Botany*. *FT* features again, now as a tool to accelerate breeding in tomatoes and potentially other crops (Bellinazzo, 2024; Deng *et al.*, 2024). In addition, for tomatoes, the other end of reproductive development: fruit ripening and the importance of gene regulatory circuits involving ethylene, is also covered (Bellinazzo, 2023; Zhao *et al.*, 2023). Finally, the old yet timeless discussions on nature versus nurture and male versus female receive attention. Regarding the former, crop resilience can be improved by taking genetic as well as environmental effects into account by studying phenotypic plasticity (Guo *et al.*, 2024; Laitinen, 2024). In terms of the latter, sex determination in plants is a complex affair, but cucurbits remain at the forefront of understanding how male versus female flowers are specified (Pelayo and Wellmer, 2024; Segura *et al.*, 2024).

Finally, if you think life is set in its ways, if you need an example of how to break free, consider the remarkable flowers of *Lacandonia*. As Paula Rudall describes in her favourite flowering image, virtually all angiosperms have flowers with the carpels in the centre, surrounded by stamens (Rudall, 2024). Not so *Lancandonia*! Here, stamens are in the centre, surrounded by carpels. Paula Rudall outlines how analysis of morphological ‘misfits’ like *Lacandonia* can be highly interesting regarding developmental constraints governing plant evolution (Rudall, 2024).

To conclude, this year’s issue encompasses flowers for beauty and for nurture; but flowers can also be intoxicating, and there is always a place for the misfits.

So whatever life hurls at you, remember that flowers and fruits are there for us at the best and at the worst of times.
